# Comparison of an oncology clinical decision-support system’s recommendations with actual treatment decisions

**DOI:** 10.1093/jamia/ocaa334

**Published:** 2021-01-31

**Authors:** Suthida Suwanvecho, Harit Suwanrusme, Tanawat Jirakulaporn, Surasit Issarachai, Nimit Taechakraichana, Palita Lungchukiet, Wimolrat Decha, Wisanu Boonpakdee, Nittaya Thanakarn, Pattanawadee Wongrattananon, Anita M Preininger, Metasebya Solomon, Suwei Wang, Rezzan Hekmat, Irene Dankwa-Mullan, Edward Shortliffe, Vimla L Patel, Yull Arriaga, Gretchen Purcell Jackson, Narongsak Kiatikajornthada

**Affiliations:** 1 Bumrungrad International Hospital, Khlong Toei Nuea, Bangkok, Thailand; 2 IBM Watson Health, Cambridge, Massachusetts, USA; 3 Columbia University, New York, New York, USA; 4 New York Academy of Medicine, New York, New York, USA; 5 Vanderbilt University Medical Center, Nashville, Tennessee, USA

**Keywords:** clinical decision-support systems, Watson for Oncology, breast cancer, lung cancer, colon cancer, rectal cancer, concordance

## Abstract

**Objective:**

IBM^(R)^ Watson for Oncology (WfO) is a clinical decision-support system (CDSS) that provides evidence-informed therapeutic options to cancer-treating clinicians. A panel of experienced oncologists compared CDSS treatment options to treatment decisions made by clinicians to characterize the quality of CDSS therapeutic options and decisions made in practice.

**Methods:**

This study included patients treated between 1/2017 and 7/2018 for breast, colon, lung, and rectal cancers at Bumrungrad International Hospital (BIH), Thailand. Treatments selected by clinicians were paired with therapeutic options presented by the CDSS and coded to mask the origin of options presented. The panel rated the acceptability of each treatment in the pair by consensus, with acceptability defined as compliant with BIH’s institutional practices. Descriptive statistics characterized the study population and treatment-decision evaluations by cancer type and stage.

**Results:**

Nearly 60% (187) of 313 treatment pairs for breast, lung, colon, and rectal cancers were identical or equally acceptable, with 70% (219) of WfO therapeutic options identical to, or acceptable alternatives to, BIH therapy. In 30% of cases (94), 1 or both treatment options were rated as unacceptable. Of 32 cases where both WfO and BIH options were acceptable, WfO was preferred in 18 cases and BIH in 14 cases. Colorectal cancers exhibited the highest proportion of identical or equally acceptable treatments; stage IV cancers demonstrated the lowest.

**Conclusion:**

This study demonstrates that a system designed in the US to support, rather than replace, cancer-treating clinicians provides therapeutic options which are generally consistent with recommendations from oncologists outside the US.

## INTRODUCTION

Oncologists and cancer-treating clinicians face a daunting task in keeping up with rapidly evolving developments in oncology. Currently, there are over 4 million citations related to cancer listed in Pubmed (www.pubmed.gov), and over 216,000 were published in 2019 alone. In addition, high patient loads resulting from a worldwide shortage of oncologists are predicted to increase in coming years.^1^ Tools are needed to help cancer-treating clinicians quickly identify relevant evidence to support informed decision making.

One tool that helps identify therapeutic options for individual patients with cancer is IBM^(R)^ Watson for Oncology (WfO). WfO is an artificial intelligence (AI)-based clinical decision-support system (CDSS).^2^ WfO incorporates National Comprehensive Cancer Network (NCCN) guidelines for cancer treatment and provides links to supporting evidence from published scientific literature.

Programs aimed at supporting individuals involved in cancer care and treatment include CancerLinQ^(R)^,^3^ Oncoanalytics^(R)^,^4^ and tools provided by Tempus^(R)^^5^; but very few formal performance evaluations of such tools have been published.^6^ WfO utilizes AI approaches, including natural language processing and machine learning, to incorporate and analyze evidence from published literature and information from NCCN guidelines and to intake individual patient information in order to provide therapeutic options to cancer-treating clinicians.^2^^,^^7–10^ CancerLinQ collects and organizes real-world data from a variety of sources within the United States (US) for use by clinicians and researchers who are involved in the care of patients with cancer. OncoAnalytics provides information to clinicians on drugs, costs of care, and billing that can help improve the delivery of cancer care to patients. Tempus facilitates precision medicine approaches through its library of clinical and molecular data that clinicians can access to help make data-driven decisions on patient care. Tools designed for use by patients that can also be accessed by clinicians include iCanDecide^(R)^^11^ and Decision Board^(R)^.^6^ iCanDecide helps breast cancer patients navigate through treatments for breast cancer, identify potential treatments, and record patient preferences, which clinicians can then access to help tailor treatment options for their patients. Decision Board provides a consultative platform to patients and their doctors that helps inform patients about treatment options and increase patient participation in treatment decision making.

Performance of a CDSS aimed at aiding clinicians in treatment decision making is often evaluated by its concordance with expert opinion or treatment decisions made in practice.^3^^,^^12^ Despite its common use, this methodology has significant limitations,^13^^,^^14^ most notably, the lack of a high-quality gold standard for accepted or preferred treatment decisions. Decisions in practice are not always optimal, which is, in part, the motivation for a CDSS. In the current study, a panel of experienced medical oncologists evaluated both the therapeutic options presented by WfO and treatment decisions for the same patients made at the point of care by cancer-treating clinicians. Consensus of the panel, blinded to the source of the paired treatment options presented, was used to determine the best treatment for patients with breast, colon, lung, and rectal cancers.

## OBJECTIVE

This study compared (1) the therapeutic options of an AI-based CDSS for oncology and (2) treatment decisions made in practice at BIH. Each was judged based on the acceptability of treatment options, using a gold standard for preferred treatment arrived at by consensus of a panel of experienced oncologists. We sought to characterize both the quality of CDSS therapeutic options and actual decisions made by cancer-treating clinicians at the point of care.

## MATERIALS AND METHODS

### Study design

**Figure 1.**
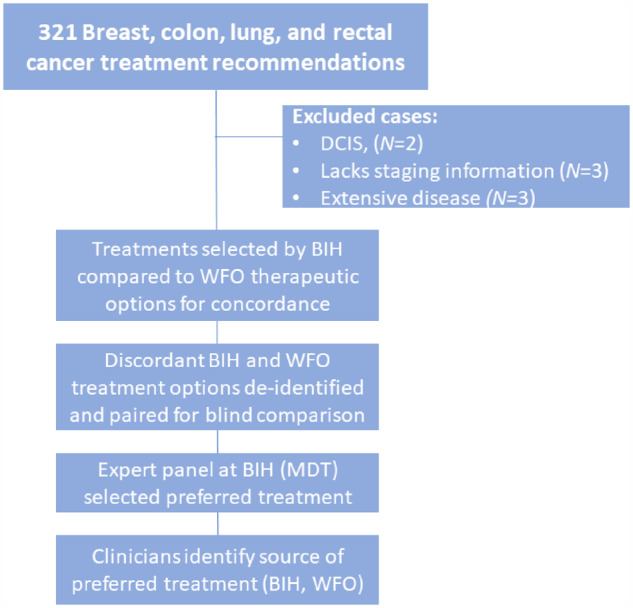
Study design. Three hundred twenty-one treatment comparisons were originally identified for inclusion in the study; excluded treatment comparisons originated from 5 cases of small-cell lung cancer, 2 cases of DCIS, and 1 case lacking staging information, resulting in 313 treatment comparisons for inclusion in this study, with characteristics summarized in Table 1.

An overview of the study design is presented in Figure 1. The study included 276 cancer patients from a diverse patient population (Supplementary Table S1) with a record of treatment at BIH between January of 2017 and July of 2018 for breast, colon, lung, and rectal cancers. We included only cases for which the therapeutic options offered by WfO were treatments that were available in Thailand at the time of treatment. The study excluded cases with either breast ductal carcinoma in situ (DCIS), small-cell lung cancer, or those that lacked staging information. Cancer stages were defined according the American Joint Committee on Cancer Staging.^15^ Bumrungrad International Institutional Review Board (BI-IRB) provided expedited approval of this prospective study under BI-IRB Project Registration Number 265-17-18 FDEK-B, approved January 24, 2019.

### Treatment evaluation

During initial treatment selection (which occurred prior study inception), clinicians treating patients for cancer did not have the opportunity to review WfO therapeutic options before selecting a treatment for their patients. The data were manually entered into WfO by 1 of 2 trained, registered nurses and validated by a board-certified oncologist; there were no reported errors in the data entry process. To evaluate the initial treatment selected and given by the clinician at BIH, as well as WfO therapeutic options for the case, research staff processed the case through WfO, and they paired treatments given by clinicians at BIH with WfO recommendations for the case, shown in green in the WfO user interface (UI). Treatments that were identical were recorded as “identical” and not reviewed further. Research staff at BIH used a code to mask the source of treatment options in the remaining nonidentical treatment pairs. The paired and coded treatment options were randomized and presented for evaluation in spreadsheet form to a panel of 4 board-certified medical oncologists from BIH. The average number of years in medical practice as a board-certified medical oncologist ranged from 10 to 45 years (mean 21.5 years).

The panel discussed and rated each of the nonidentical treatment pairs by consensus as either both acceptable and roughly equivalent; both acceptable, but 1 preferred; 1 acceptable and the other unacceptable; or both unacceptable. Acceptability was defined by the panel as compliant with BIH institutional practices, and unacceptability was defined as noncompliant with BIH institutional practices. Each of the 3 medical oncologists on the panel independently evaluated treatment options, and discrepancies were resolved by consensus. The code used for the blinding procedure allowed research staff to link treatment options evaluated to the source of each option (BIH or WfO) after the panel evaluations were complete.

### Clinical Decision-Support system

The WfO system operation has been described^2^^,^^7–10^ (see supplement to Somashekhar et al).^2^ Briefly, the system contains a set of training cases that serve as the source of ground truth for the system. In the case of breast cancer, the breast cancer module contains a repository of 270 attributes that were verified by experts as evidence-supported attributes needed for personalized treatment decisions. Examples include family medical history, comorbidities, functional status, endocrine status, genetic profiling, tumor characteristics, prior treatment modalities and major organ function status. The system provides treatment recommendations for a given case using the attributes that are relevant to each case. The knowledge base contains an extensive corpus of oncology journals and peer-reviewed publications, as well as NCCN guidelines and other reliable sources, that can be searched for evidence that matches a particular case. WfO processes text documents using machine learning and natural language processing, to enable identification of articles from the literature that match specific patient characteristics and the therapies for consideration.^2^^,^^7–10^ An in-depth description of the system can be found in the supplemental information to Somashekhar et al.^2^

WfO offers ranked treatment options to clinicians and can provide links to associated NCCN guidelines and supporting evidence. The UI offers an option to explicitly include NCCN guidelines in the evidence for each choice. The therapeutic options are presented as ‘recommended’ in green, ‘for consideration’ in yellow, and ‘not recommended’ in red. Options that are categorized as ‘recommended’ and ‘for consideration’ are both considered acceptable therapeutic options by WfO. In this study, the treatment selected by BIH was paired with the therapeutic option shown in green in the UI that was also a treatment available to patients in Thailand. In cases with more than 1 recommended WfO option in green that disagreed with BIH treatment, these treatment pairs were not evaluated, as multiple comparisons of the same case could potentially facilitate inadvertent identification of the source of treatment option by the panel. A match between the choice of treatment by BIH and any of WfO’s recommended therapeutic options (shown in green in the UI) was recorded as identical and not reviewed further by the panel.

### Statistical methods

Descriptive statistics were used to summarize the clinical and demographic characteristics of the study population by cancer type and stage. Frequency and proportions were calculated across 7 treatment decision categories by cancer type and stage. Differences in unacceptable treatment options between BIH and WfO were determined using chi-square or Fisher's exact test where appropriate. All analyses were performed on RStudio with Open Source R version 3.5.2.

## RESULTS

### Study population

We identified a total of 276 patients that were treated during the study period for breast, colon, rectal, or lung cancers, ranging in age from 24 to 94, with a median age of 60; the nationality and country of origin of these patients are shown in Supplementary Table S1. Of this group, a total of 8 cases were excluded: 5 cases of small cell lung cancer, 2 cases of DCIS, and 1 case that lacked staging information. Because WfO sometimes offers more than 1 recommended therapeutic option, this resulted in 313 treatment pairs for evaluation, which included 126 breast, 70 colon, 29 rectal, and 88 lung paired treatments (Figure 1 and Table 1). The study included a relatively larger number of breast cancer treatment pairs (126) as compared to lung, colon, or rectal cancer (88, 70, and 29, respectively). When combining all 4 cancers together, there was a greater number of treatment pairs related to stage IV disease (117), as compared to stages I–III (80, 67, and 49, respectively).

**Table 1.** Treatment decisions

Type or Stage		*N*	Age (Median)
**Stage I-IV (*N *=* *313)**	Breast	126	52
	Colon	70	64
	Lung	88	65.5
	Rectal	29	56
**Breast (*N *=* *126)**	stage I	56	54
	stage II	37	58
	stage III	10	41
	stage IV	23	44
**Colon (*N *=* *70)**	stage I	9	63
	stage II	16	67.5
	stage III	24	61.5
	stage IV	21	64
**Lung (*N *=* *88)**	stage I	10	65.5
	stage II	8	72
	stage III	3	74
	stage IV	67	65
**Rectal (*N *=* *29)**	stage I	5	52
	stage II	6	61.5
	stage III	12	55.5
	stage IV	6	48

### Evaluations of treatment options for all cancer types and stages combined

**Figure 2.**
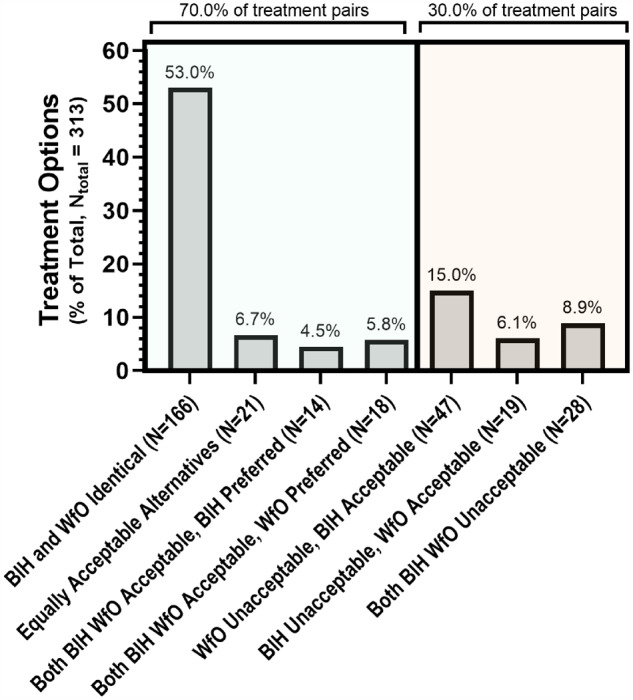
Panel evaluations of treatment pairs as a percentage of all cancer types and stages combined. Evaluation of treatment pairs from comparison of WfO therapeutic options to treatments recommended at the point of care by cancer-treating clinicians at BIH are shown as a percent of total (*N*_total_ = 313). Identical treatments or those that the panel found to be acceptable alternatives are shaded green; decisions that were not in agreement between BIH and WfO that were also unacceptable to the panel, with respect to either or both BIH and WfO, are shaded orange.

Results of treatment evaluations for all cancer types and stages combined are shown in Figure 2. Identical treatments were noted as such and did not undergo further review by the panel. Overall, 70% (219) of treatment options offered by WfO were found to be acceptable by the panel, with 59.7% (187) of WfO options identical to, or rated equally acceptable to, BIH treatments. Of the 94 evaluations in which 1 or both nonidentical treatment options were found to be unacceptable by the panel (Table 2), 19 treatments offered by BIH were unacceptable with respect to BIH (20.2%), 47 were unacceptable with respect to WfO (50%), and 28 were unacceptable with respect to both BIH and WfO (29.8%, Table 3).

**Table 2.** Aggregated results, grouped by cancer type and stage

		Identical or Acceptable Alternatives	Either or Both Unacceptable
		*N* (%)	*N* (%)
By cancer type, all stages combined	Breast (N = 126)	75 (59.3)	51 (40.5)
	Colon (N = 70)	59 (84.3)	11 (15.7)
	Lung (N = 88)	60 (68.2)	28 (31.8)
	Rectal (N = 29)	25 (86.2)	4 (13.8)
	N_total_ =313	219	94
By stage, all cancers combined	Stage I (N = 80)	56 (70)	24 (30)
	Stage II (N = 67)	49 (73.1)	18 (26.9)
	Stage III (N = 49)	40 (81.6)	9 (18.4)
	Stage IV (N = 117)	74 (63.2)	43 (36.8)
	N_total_ =313	219	94

**Table 3.** Distribution of acceptable vs unacceptable treatment options

Cancer Types (Stage) (N_total_ = 313)	Identical or Acceptable Alternatives (*N *=* *219 cases)					Either or Both Unacceptable (*N *=* *94 cases)			
	Identical	WfO, BIH Equally Acceptable	Both Acceptable, BIH Preferred	Both Acceptable, WfO Preferred	*N* (%)	BIH Unacceptable	WfO Unacceptable	Both Unacceptable	*N* (%)
Breast, (I)	22	4	1	6	33 (58.9)	2	12	9	23 (41.1)
Breast, (II)	15	6	0	4	25 (67.6)	8	2	2	12 (32.4)
Breast, (III)	5	3	0	0	8 (80.0)	2	0	0	2 (20.0)
Breast, (IV)	2	2	4	1	9 (39.1)	3	4	7	14 (60.9)
Breast, (I-IV)	44	15	5	11	75 (59.5)	15	18	18	51 (40.5)
Colon, (I)	9	0	0	0	9 (100.0)	0	0	0	0 (0.0)
Colon, (II)	12	1	1	1	15 (93.8)	0	0	1	1 (6.3)
Colon, (III)	19	0	2	0	21 (87.5)	0	2	1	3 (12.5)
Colon, (IV)	14	0	0	0	14 (66.7)	0	6	1	7 (33.3)
Colon, (I-IV)	54	1	3	1	59 (84.3)	0	8	3	11 (15.7)
Lung, (I)	8	0	0	1	9 (90.0)	0	0	1	1 (10.0)
Lung, (II)	4	0	1	0	5 (62.5)	0	3	0	3 (37.5)
Lung, (III)	1	0	0	0	1 (33.3)	0	2	0	2 (66.7)
Lung, (IV)	35	1	4	5	45 (67.2)	4	14	4	22 (32.8)
Lung, (I-IV)	48	1	5	6	60 (68.2)	4	19	5	28 (31.8)
Rectal, (I)	5	0	0	0	5 (100.0)	0	0	0	0 (0.0)
Rectal, (II)	3	1	0	0	4 (66.7)	0	1	1	2 (33.3)
Rectal, (III)	9	1	0	0	10 (83.3)	0	1	1	2 (16.7)
Rectal, (IV)	3	2	1	0	6 (100.0)	0	0	0	0 (0.0)
Rectal, (I-IV)	20	4	1	0	25 (86.2)	0	2	2	4 (13.8)

### Evaluation of treatment options, aggregated by cancer type (all stages) or stage of cancer (all types)

Table 2 shows the aggregate results of treatment-decision comparisons grouped by cancer type and stage. When examining evaluation of treatment options by cancer type (all stages combined), agreement between WfO and BIH treatment options (identical or acceptable alternatives) was lowest for breast cancer (59.3%, *N *=* *75), as compared to rectal (86.2%, *N *=* *25), colon (84.3%, *N *=* *59), or lung cancer (68.2%, *N *=* *60). Rectal cancer had the highest percentage of identical or acceptable treatments, followed by colon cancer. Combining all 4 cancer types, stage IV had the lowest proportion of identical or acceptable alternatives (63.2%, *N *=* *74), with a relatively greater proportion of identical or acceptable alternatives for stages I (70%, *N *=* *56), II (73.1%, *N *=* *49), and III (81.6%, *N *=* *40 cases, Table 2).

After combining all stages of colon cancer, there were significantly more (*P* = .01) unacceptable therapeutic options originating from WfO (*N* = 8, 11.4%) than BIH (0, 0%). Similarly, combining all stages of lung cancer, we found significantly more (*P* = .002) unacceptable options from WfO (*N* = 19, 21.6%) than BIH (4, 4.5%). After combining all cancer types, we found significantly more (*P* = .01) unacceptable options from WfO for stage I cancer (*N* = 12, 15%) than BIH (*N* = 2, 2.5%). Likewise, for stage IV of all cancer types combined, there were significantly more (*P* = .002) unacceptable options from WfO (*N* = 24, 20.5%) than BIH (*N* = 7, 6.0%).

### Identical or equally acceptable treatment options of each cancer type by stage

**Figure 3.**
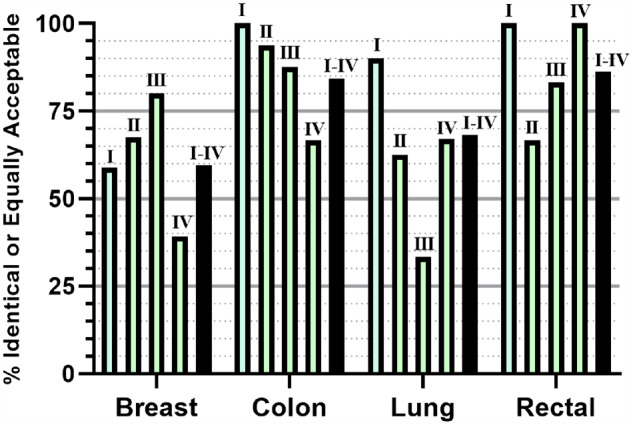
Identical or equally acceptable treatment pairs by cancer type and stage. Identical or equally acceptable options are shown as a percent of total treatment comparisons for each cancer type (green bars), or as percent of all stages combined for the indicated cancer type (black bars). Stage is indicated by Roman numeral above each bar. Breast, *N *=* *126; colon, *N *=* *70; lung, *N *=* *88; rectal, *N *=* *29. Stage I, *N *=* *80; stage II, *N *=* *67; stage III, *N *=* *49; stage IV, *N *=* *117; *N*_total_ = 313.

When examining concordance of breast cancer options by stage, we found that the percentage of identical and equally acceptable options tended to increase from stage I to stage III, with a sharp decline in agreement for stage IV breast cancer (Figure 3). For colon cancer, identical and equally acceptable options were greatest for early-stage cancer and tended to decrease sequentially for stages II–IV. For lung and rectal cancers, stages I and IV showed the highest percentage of identical or acceptable options. The black bars in Figure 3 show the mean agreement for all stages combined for each cancer type.

### Comparison of treatment options by cancer stage and type

Treatment option evaluations by cancer stage and type are compared in Table 3. In the 32 treatment pairs where differing WfO and BIH options were both acceptable to the panel but 1 option preferred, WfO was preferred 18 times and BIH 14 times. The proportion of unacceptable treatment options originating from either or both BIH or WfO was highest for breast cancer and relatively low for early stage lung cancer, as well as most stages of colon and rectal cancer, consistent with the greater proportion of acceptable treatment option alternatives for colorectal cancers than other cancer types (Table 3).

Although reasons for discordance were not collected in this study, Table 4 lists several examples where either 1 or both therapies were found to be unacceptable, as well as cases where 1 therapy was preferred over another, according to notes available from the panel discussion.

**Table 4.** Examples of therapy choices made by panel

Panel Rating	Both BIH and WfO Acceptable, BIH Preferred	Both BIH and WfO Acceptable, WfO Preferred	WfO Unacceptable, BIH Acceptable	BIH Unacceptable, WfO Acceptable	Both BIH and WfO Unacceptable
	Example 1	Example 2	Example 3	Example 4	Example 5
**Reason for panel's choice of therapy**	BIH chemotherapy has lower kidney toxicity given patient age.	WfO targeted therapy is viewed as more effective by panel	WfO chemotherapy toxicity unacceptable for patient age	Patient should receive chemotherapy	Both treatments are out of date for stage III rectal cancer
**Case**	Lung cancer, stage IIB age 75 years	Lung cancer, stage IV	Lung cancer, stage IIB, age 75 years	Lung cancer, stage IV, age 70 years	Rectal cancer, stage IIIB
**WfO therapy**	Cisplatin/Paclitaxel with concurrent conventional radiation therapy	Osimertinib	Cisplatin/Etoposide with concurrent conventional radiation therapy	Cisplatin/Pemetrexed + Bevacizumab	Fluorouracil/leucovorin followed by infusional Fluorouracil and radiation therapy
**BIH therapy**	Carboplatin/Paclitaxel with concurrent conventional radiation therapy	Gefitnib	Carboplatin/Paclitaxel with concurrent conventional radiation therapy	Palliative radiation	Infusional Fluorouracil and radiation therapy
	Example 6	Example 7	Example 8	Example 9	Example 10
**Reason for panel's choice of therapy**	BIH chemotherapy containing oxaliplatin was viewed as more effective by panel	WfO targeted therapy was more affordable	Bevacizumab usage introduces bleeding risk to patient, not as affordable as BIH option	Chemotherapy needed to treat large tumor	Patient unable to tolerate systemic therapy
**Case**	Colon cancer, stage IIIB	Lung cancer, stage IV	Lung cancer, stage IV	Breast cancer, stage IIB	Breast cancer, stage IV ECOG^a^ 3
**WfO therapy**	Fluorouracil/Leucovorin	Alectinib	Cisplatin/Pemetrexed + Bevacizumab	Doxorubicin/Cyclophosphamide followed by Paclitaxel, then Letrozole and radiation therapy	Fulvestrant + Palbociclib
**BIH therapy**	Capecitabine/Oxaliplatin	Crizotinib	Carboplatin/Pemetrexed	Exemestane with no radiation therapy	Eribulin

a ECOG, Eastern Cooperative Oncology Group, scale to assess cancer patient functional status.

## DISCUSSION

This study is one of the first evaluations of an AI-based CDSS for oncology that not only assesses the quality of the CDSS therapeutic options offered but also compares them to treatment decisions that were made by cancer-treating clinicians. WfO, developed in the US, incorporates best-practice recommendations provided by NCCN guidelines.^2^^,^^7^^,^^9^^,^^10^^,^^13^ Our evaluation shows the potential for a CDSS that is developed in the US to provide acceptable therapeutic options for patient populations outside the US. In more than two-thirds of the treatment comparisons, both treatment options were found to be acceptable providing evidence that, in many cases, the CDSS performed at the level of BIH’s experienced panel. In cases where one acceptable option was preferred over another acceptable option, the panel’s preference was split relatively equally between WfO therapeutic options and treatment decisions made in practice. In cases where one option was unacceptable to the panel, the WfO treatment option was more often viewed as unacceptable, compared to treatments selected by clinicians at BIH. These findings illustrate the role for CDSS in supporting, rather than replacing, clinician decision making.

Individual clinicians, working together with the CDSS, may have the potential to perform better than either would alone. Supporting this idea, one study demonstrating the use of WfO in a multidisciplinary tumor board setting for high-risk breast cancer cases resulted in changed treatment decisions in 5% of cases and increased guideline adherence from 89% to 97%.^16^^,^^17^ However, it is also possible that use of a CDSS may increase time, present outdated information, or be ignored by clinicians. More studies are needed to evaluate the extent to which advice of the CDSS would inform a clinician’s ultimate treatment decision. This, along with a complete listing of reasons for panel disagreement with therapeutic options, may help inform development and refinement of the CDSS.

There were cases where both WfO and BIH treatments were unacceptable to the panel, most often in stage IV lung cancer and most stages of breast cancer. This may be due, in part, to changes in institutional practices in the interim between patient treatment in 2017–2018 and the evaluation of those treatments in 2019, including treatments that incorporate precision oncology^18^ and newly approved systemic therapies, such as targeted drugs,^19^^,^^20^ immunotherapies,^21^^,^^22^ and antibody-drug conjugates. Consistent with this idea, we present several examples where the panel preferred targeted therapy over earlier therapies used to treat stage IV lung cancer patients at the point of care, reflecting the rapid evolution in the therapeutic landscape for metastatic lung cancer.

Care and treatment of advanced disease can be influenced by a number of factors, including treatment preferences by patients, providers, as well as geographic treatment preferences. Patient comorbidities, cultural practices, and financial and quality of life considerations can also play important roles in treatment decision making by clinicians, patients, and their families. Accordingly, we recognize that WfO’s recommendations in all cases, but especially in complex stage IV settings, should be viewed as suggestions rather than mandates. We acknowledge that because of the breadth of cancers included in this study, the size of some of the cancer subgroups are too small to draw definitive conclusions. Thus, results of data presented herein should be interpreted with caution, due to the inadequacy of power for subgroup analysis.

Treatment options for colorectal cancers had the highest proportion of identical or acceptable alternatives. Despite the relatively high agreement for colorectal cancers in general, there was at least one case where the panel found both WfO and BIH alternatives to be out of date by the time the panel reviewed the case. It is also important to note that WfO therapeutic suggestions are based on treatments available in the US. Some of the WfO treatment options may not be available or financially feasible in other countries, which would likely be reflected in a reduced concordance as compared to those treatments that are more widely available.

In this work, local medical oncologists, well-versed in best practices for cancer treatment and standards of care for their patient population, objectively evaluated the performance of the CDSS through blind comparisons of therapeutic options offered by the CDSS to treatment decisions made in practice. Although agreement with individual practice decisions is often the metric employed in CDSS evaluations,^14^ such concordance studies can be difficult to interpret.^23^ Individual experts often differ in what they believe the best course of treatment may be for a particular patient.^24^ Blinding is a standard way to minimize confirmation bias, which may result from a preference for one treatment over another, based on preconceived ideas and beliefs.^25^ This type of bias can be either a conscious, explicit belief, or an implicit, unconscious belief on the part of an evaluator regarding the best origin of treatment decisions. While this approach is almost uniformly applied in interventional trials,^26^ it is not often adopted in the comparison of human beings and CDSSs that are designed to support cancer-treating physicians.

## CONCLUSION

This study, which compared treatment decisions made by individual clinicians and therapeutic options offered by an oncology CDSS, demonstrated agreement in the majority of cases, a relatively equal number of cases for which clinician decisions or CDSS options were favored by an experienced panel, and some cases for which both were considered unacceptable. These findings illustrate the fact that humans in practice do not always choose the best course of treatment, identifying a gap where CDSS could improve performance. Blinded therapeutic evaluation studies are a reasonable *first step* in measuring technical performance of CDSS and a baseline of human performance.^17^ Because most CDSSs are intended to augment rather than replace clinician decision making, a comparison of a CDSS alone versus clinician may not be an appropriate way to evaluate the system’s intended use. Instead, future studies should examine the quality of decisions by clinicians, with and without support from the CDSS, and ultimately determine value with long-term health outcomes studies.

## FUNDING

This work was funded by IBM Watson Health.

## AUTHOR CONTRIBUTIONS

AMP conceived, drafted, and finalized the manuscript; SS, HS, TJ, SI, NT, PL, WD, WB, NT, PW, and KN designed the study and collected data for the manuscript; SW analyzed data; all authors contributed to interpretation of the data and approval of the final version.

## SUPPLEMENTARY MATERIAL

Supplementary material is available at *Journal of the American Medical Informatics Association* online.

## DATA AVAILABILITY

The data underlying this article cannot be shared publicly due to privacy concerns of the individuals that participated in the study.

## Supplementary Material

ocaa334_Supplementary_Data
