# Supplementary material for: Comparison of an oncology clinical decision-support system’s recommendations with actual treatment decisions
Source: J Am Med Inform Assoc. 2021 Jan 31;28(4):832–8. doi: 10.1093/jamia/ocaa334 (PMC7973455; doi:10.1093/jamia/ocaa334)
Supplement: ocaa334_Supplementary_Data [file ocaa334_supplementary_data.docx]

| **Supplementary Table S1: Country of Residence and Nationality (N = 276)** | | | |
| --- | --- | --- | --- |
| **Country of Residence** | **Number of cases** | **Nationality** | **Number of cases** |
| Bangladesh | 11 | AfricanSouth | 1 |
| Cambodia | 10 | American | 3 |
| China | 5 | Bangladeshi | 10 |
| Eritrea | 1 | Belgium | 1 |
| Ethiopia | 7 | British | 3 |
| Indonesia | 1 | Cambodian | 10 |
| Iran | 1 | Canadian | 1 |
| Kuwait | 11 | Chinese | 6 |
| Lao | 1 | Emirate | 2 |
| Mongolia | 2 | Eritrean | 1 |
| Myanmar | 59 | Ethiopian | 7 |
| Nepal | 1 | Filipino | 1 |
| Oman | 22 | Hungary | 1 |
| Qatar | 6 | Indian | 1 |
| Sudan | 4 | Indonesia | 1 |
| Thailand | 108 | Iran | 1 |
| United Arab Emirates | 18 | Japan | 1 |
| United States of America | 1 | Japanese | 1 |
| Vietnam | 7 | Kuwaiti | 10 |
|  |  | Malaysian | 1 |
|  |  | Mongolia | 2 |
|  |  | Morocco | 1 |
|  |  | Myanmar | 59 |
|  |  | Nepal | 1 |
|  |  | Omani | 22 |
|  |  | Qatari | 6 |
|  |  | Russian Federation | 1 |
|  |  | Sudanese | 4 |
|  |  | Sweden | 1 |
|  |  | Swedish | 1 |
|  |  | Syrian | 1 |
|  |  | Syrian Arab Republic | 1 |
|  |  | Thai | 94 |
|  |  | United Arab Emirates | 10 |
|  |  | Vietnamese | 6 |
|  |  | Yemeni | 3 |
